# Estrogen Receptor and PI3K/Akt Signaling Pathway Involvement in *S-*(-)Equol-Induced Activation of Nrf2/ARE in Endothelial Cells

**DOI:** 10.1371/journal.pone.0079075

**Published:** 2013-11-19

**Authors:** Ting Zhang, Xinyu Liang, Linying Shi, Li Wang, Junli Chen, Chao Kang, Jundong Zhu, Mantian Mi

**Affiliations:** Research Center for Nutrition and Food Safety, The Third Military Medical University, Chongqing, PR China; Goethe University, Germany

## Abstract

*S-*(-)equol, a natural product of the isoflavone daidzein, has been reported to offer cytoprotective effects with respect to the cardiovascular system, but how this occurs is unclear. Interestingly, *S-*(-)equol is produced by the human gut, suggesting a role in physiological processes. We report that treatment of human umbilical vein endothelial cells and EA.hy926 cells with *S-*(-)equol induces ARE-luciferase reporter gene activity that is dose and time dependent. *S-*(-)equol (10–250 nM) increases nuclear factor-erythroid 2-related factor 2 (Nrf2) as well as gene products of Nrf2 target genes heme oxygenase-1 (HO-1) and NAD(P)H (nicotinamide-adenine-dinucleotide-phosphate) quinone oxidoreductase 1 (NQO1). Endothelial cells transfected with an HA-Nrf2 expression plasmid had elevated HA-Nrf2, HO-1, and NQO1 in response to *S-*(-)equol exposure. *S-*(-)equol treatment affected Nrf2 mRNA only slightly but significantly increased HO-1 and NQO1 mRNA. The pretreatment of cells with specific ER inhibitors or PI3K/Akt (ICI182,780 and LY294002) increased Nrf2, HO-1, and NQO1 protein, impaired nuclear translocation of HA-Nrf2, and decreased ARE-luciferase activity. Identical experiments were conducted with daidzein, which had effects similar to *S-*(-)equol. In addition, DPN treatment (an ERβ agonist) induced the ARE-luciferase reporter gene, promoting Nrf2 nuclear translocation. Cell pretreatment with an ERβ antagonist (PHTPP) impaired *S-*(-)equol-induced Nrf2 activation. Pre-incubation of cells followed by co-treatment with *S-*(-)equol significantly improved cell survival in response to H_2_O_2_ or tBHP and reduced apoptotic and TUNEL-positively-stained cells. Notably, the ability of *S-*(-)equol to protect against H_2_O_2_-induced cell apoptosis was attenuated in cells transfected with an siRNA against Nrf2. Thus, beneficial effects of *S-*(-)equol with respect to cytoprotective antioxidant gene activation may represent a novel strategy to prevent and treat cardiovascular diseases.

## Introduction

Soy isoflavones (genistein and daidzein) have been reported to restore endothelial function after diverse injuries (atherosclerosis, menopause, diabetes mellitus or smoking) [1]. However, *in vivo* data provide contradictory information about this activity. Equol, a specific metabolic product of daidzein, exists in two forms: *S-*(-)equol and *R-*(+)equol, and synthetic equol is a racemic mixture. Only *S-*(-)equol occurs naturally, it is produced in the gut of people with a particular metabolic phenotype (30–50% of the population). Almost half of the plasma concentration of equol (49.7%) circulates in the free form, in contrast to its precursor, daidzein (18.7%) [2]. Equol also has the greatest antioxidant activity of all identified isoflavones [2], so antioxidant activities of its individual enantiomers should be similar. However, *S-*(-)equol binds with higher affinity to ERβ than *R-*(+)equol or daidzein and the *S-*form is more effective at triggering gene transcription [3]. Therefore, investigations into the pharmacology of equol have correlated plasma and urine concentrations to explain soy isoflavone health benefits in humans. Specifically, people who can produce *S-*(-)equol may benefit more from soy isoflavone intake than those who cannot perform this endogenous task.

Equol has been reported to be of benefit in atherogenesis, cancer, diseases of the nervous system, symptoms or menopause, and osteoporosis, among other conditions [4]. To address the cardiovascular protective effects of *S-*(-)equol, a randomized controlled study suggested that hypercholesterolemic adults whose diets included 80 g/d of isoflavone-enriched pasta (33 mg/serving) had significantly decreased total serum cholesterol, LDL-cholesterol, and C-reactive protein compared to individuals who consumed the same amount of pasta without isoflavones [5]. Moreover, “equol producers” had significantly less LDL-cholesterol and C-reactive protein compared with “non-equol producers”. Multiple mechanisms may explain how soy, soy isoflavones, and *S-*(-)equol contribute to health, including lowering total and LDL cholesterol, increasing HDL cholesterol, decreasing CRP, improving endothelial function, decreasing the reactive oxygen species (ROS) production, and so forth. Other mechanisms reported in the literature to explain the action of *S-*(-)equol include inhibition of MEK, activation of eNOS and AMPK, activation of cellular antioxidant defense mechanisms and direct antioxidant effects to remove free radicals. However, in virtually all of these studies, racemic equol was used. Thus, the mechanism by which individual enantiomers, such as *S-*(-)equol, provide cardiovascular benefits is unclear.

Nrf2 (Nuclear factor-erythroid 2-related factor 2) is an important sensor of oxidative and electrophilic stress, ensuring the maintenance of redox homeostasis by regulating transcriptional activation of phase II defense and antioxidant genes [6]. Patients with cardiovascular diseases have elevated ROS, which contribute to impaired endothelial function and reduced bioavailability of nitric oxide [7]. Heme oxygenase-1 (HO-1) is a cardioprotective antioxidant enzyme, which is often overexpressed in atherosclerotic lesions and may be regulated by Nrf2 [8]. Nrf2 also induces the expression of the antioxidant-defense enzyme NAD(P)H (nicotinamide-adenine-dinucleotide-phosphate) quinone oxidoreductase 1 (NQO1). Although the effect of equol on Nrf2 has been examined in hepatic cells, a similar analysis in vascular cells has not been reported [9]. Equol is a known estrogen receptor β (ERβ) activator [4], and could rapidly stimulated nitric oxide synthase (eNOS) accumulation in endothelial cells [10]. NO arising from enhanced eNOS synthesis inhibits modified LDL formation and increases NAD(P)H oxidase [1], [11], [12], [13]. In response to NO, Nrf2 undergoes translocation to the nucleus to bind the antioxidant response element (ARE) of antioxidant genes and facilitate transcription. The direct relationship between the ER and the Nrf2/ARE signaling pathway is, however, unknown. PI3K/Akt signal transduction systems are involved in a number of cellular processes, including cell proliferation and cell death. Moreover, factors upstream of Nrf2 and ARE-genes in the *S-*(-)equol (genistein and daidzein) response pathway and the mechanism by which *S-*(-)equol exerts its antioxidant effect warrant investigation. Additionally, the benefit of soy isoflavones in the treatment of cardiovascular disease has been controversial [14]. Thus, we addressed whether *S-*(-)equol activates Nrf2 in endothelial cells and examined the involvement of ER and PI3K/Akt pathways in the activation of Nrf2/ARE after *S-*(-)equol (daidzein) treatment. We also studied the role of Nrf2 in the antioxidant response induced by *S-*(-)equol in endothelial cells injured by oxidative stress.

## Results

### Identification of *S*-(-)Equol as an Nrf2 Activator

**Figure 1:**
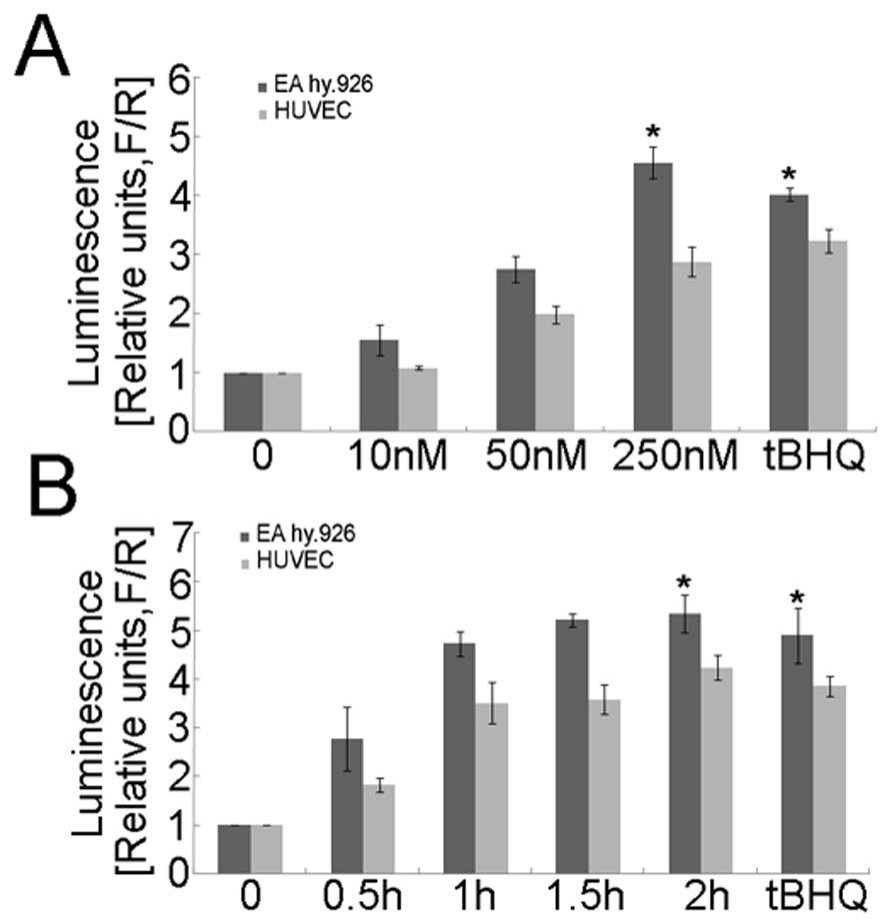
Identification of *S-*(-)equol as an Nrf2 activator. Where indicated, EA.hy926 cells or HUVECs were transfected with plasmids containing an ARE-dependent firefly luciferase reporter gene (*F*) and the renilla firefly luciferase gene (*R*), the latter of which was included in each transfection reaction as a control for transfection efficiency. Medium was changed after 6 hours of incubation. (A) Cells were treated with *S-*(-)equol (10 nM, 50 nM, and 250 nM) or tBHQ (50 µM) for 16 h. (B) Cells were treated with *S-*(-)equol (250 nM) and tBHQ (50 µM) for the time periods indicated. The potency of induction is expressed as the relative luminescence (R/F) of the treated samples over the untreated transfected controls (mean ± SD, n = 3). *p<0.05.

To determine whether *S-*(-)equol treatment activates Nrf2 in endothelial cells, we assayed the effect of *S-*(-)equol on the expression of a previously described NQO1-ARE-dependent firefly luciferase reporter gene [15]. The activity of the NQO1-ARE-dependent firefly luciferase reporter was measured in parallel with a renilla luciferase construct, which was used as an internal control for transfection efficiency and a reference sample for assaying *S-*(-)equol toxicity. tBHQ, a known Nrf2-activator, was used as a positive control. *S-*(-)equol treatment resulted in a dose-dependent increase in ARE-dependent luciferase activity (Figure 1A). The induction (1.52-fold in EA.hy926 cells and 1.08-fold in HUVEC) of the reporter gene was observed at an *S-*(-)equol concentration as low as 10 nM and reached a maximum (4.49-fold in EA.hy926 cells and 2.88-fold in HUVEC) at 250 nM. *S-*(-)equol-induced increases in ARE-dependent luciferase activity were also time dependent (Figure 1B). Time points of less than 2 h were used for subsequent experiments because Nrf2 and NOQ1 could be readily detected within this time frame.

### *S*-(-)Equol Increases Nrf2 Protein, which in Turn Enhances ARE-dependent Transcription

**Figure 2:**
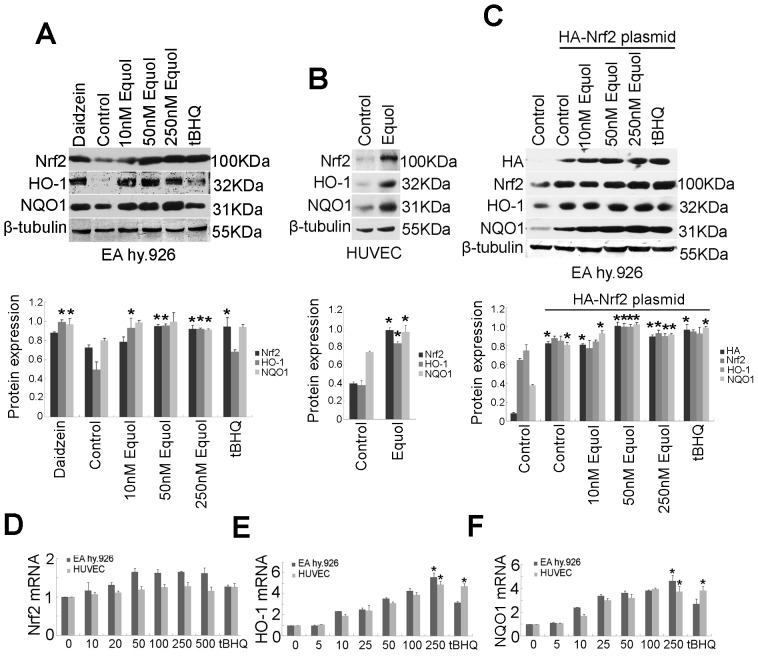
*S-*(-)equol primarily up-regulated Nrf2 protein expression activating ARE-dependent response. (A) Whole EA.hy926 cells lysates of cells treated with 250 nM *S-*(-)equol, 500 nM daidzein, or 50 µM tBHQ for 24 h were subjected to immunoblot analysis with anti-Nrf2, anti-HO-1, anti-NQO1, and anti-β-tubulin antibodies. (B) Total HUVECs lysates of cell treated with 250 nM *S-*(-)equol for 24 h were immunoblotted with anti-Nrf2 and anti-β-tubulin antibodies. (C) EA.hy926 cells were transfected with plasmids containing an HA-tagged Nrf2 open reading frame and the renilla firefly luciferase gene. Medium was changed after 6 hours of incubation. Where indicated, extracts from HA-Nrf2 EA hy.92 cells were additionally probed with anti-HA antibody, anti-Nrf2, anti-HO-1, anti-NQO1 and anti-β-tubulin antibodies. Values are means of three independent experiments with standard deviations represented by vertical bars. Mean values were significantly different compared to controls (*p<0.05). Total RNA was extracted from EA.hy926 cells or HUVECs treated as indicated, and Nrf2 (D), HO-1 (E), and NQO1 (F) mRNA was measured by real-time RT-PCR analysis. The values shown represent the mean ± SD obtained for three independent experiments (*p<0.05).

According to Zhang and colleagues, the Nrf2 signaling pathway is mainly regulated by the inhibition of Nrf2 protein degradation, which is mediated by Keap1-dependent ubiquitin conjugation [16]. Here, we report that *S-*(-)equol treatment causes a dose-dependent increase in Nrf2 and a concomitant increase in protein products of the Nrf2 target genes HO-1 and NQO1 (Figure 2A) in EA.hy926 cells. The soy isoflavone daidzein produces similar effects (Figure 2A). We also found that *S-*(-)equol up-regulated Nrf2, HO-1, and NQO1 protein in HUVECs (Figure 2B). In a previous study, we discovered that treatment of EA.hy926 cells with genistein for 1 to 16 hrs increased Nrf2 but this was not dose-dependent. To corroborate the finding that increased Nrf2 correlates with ARE-dependent gene activation, we measured HO-1 and NQO1 protein in EA.hy926 cells transfected with an HA-Nrf2 expression plasmid. Cells treated with *S-*(-)equol had increased Nrf2 protein, and HO-1 and NQO1 were increased in cells overexpressing HA-Nrf2 (Figure 2C). The induction of the Nrf2 pathway by activators such as tBHQ mainly involves the maintenance of Nrf2 protein levels and not Nrf2 transcript upregulation. We therefore investigated the effects of *S-*(-)equol on Nrf2, HO-1, and NQO1 mRNA using real-time RT-PCR. *S-*(-)equol treatment resulted in a slight, but not significant, dose-dependent increase in Nrf2 mRNA (Figure 2D) and caused significant dose-dependent increases in HO-1 and NQO1 mRNA (Figure 2E and F) both in EA.hy926 cells and HUVECs. These results suggest that *S-*(-)equol activates the Nrf2/ARE pathway primarily through the post-transcriptional regulation of Nrf2 protein.

### Involvement of the ER and PI3K/Akt Pathways in Nrf2 Activation Induced by *S*-(-)Equol Exposure

**Figure 3:**
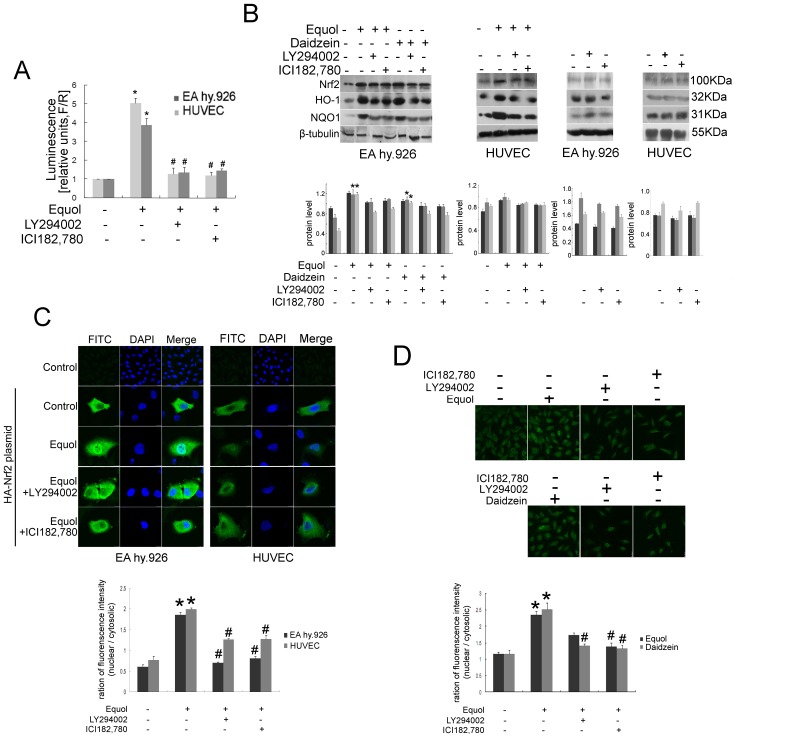
The effects of PI3K/Akt and ER inhibitors on *S-*(-)equol-induced Nrf2 activation in endothelial cells. (A) EA.hy926 cells or HUVECs were transfected with ARE-dependent firefly luciferase reporter gene (*F*) and the renilla firefly luciferase gene (*R*), and treated with 250 nM *S-*(-)equol in the presence or absence of 10 µM LY294002 or 100 nM ICI182,780 for 16 h. The potency of induction is expressed as the relative luminescence (R/F) measured using the dual luciferase reporter assay system (mean ± SD, n = 3). *p<0.05 versus with control group, ^#^ *p*<0.05 versus with *S-*(-)equol treated group. (B) EA.hy926 cells or HUVECs were incubated with or without 10 µM LY294002 or 100 nM ICI182,780 for 30 min and then with or without 250 nM *S-*(-)equol or 500 nM daidzein for 24 h. Whole cell lysates were then immunoblotted with antibodies against Nrf2, HO-1, NQO1, and β-tubulin. Values are means of three independent experiments with standard deviations represented by vertical bars. Mean values were significantly different compared with controls (*p<0.05). (C) EA.hy926 cells or HUVECs cotransfected with the HA-Nrf2 and renilla firefly luciferase expression plasmids were treated with 250 nM *S-*(-)equol in the presence or absence of 10 µM LY294002 or 100 nM ICI182,780 for 16 h, and HA-Nrf2 localization was analyzed by confocal microscopy. (D) EA.hy926 cells were incubated with 250 nM *S-*(-)equol, 500 nM daidzein with or without 10 µM LY294002 or 100 nM ICI182,780 for 16 h and then immunostained with an antibody against Nrf2, and Nrf2 localization was analyzed by confocal microscopy. Values (intensity of nuclear versus cytoplasmic) are means of counting 100 cells with standard deviations represented by vertical bars. *p<0.05 versus with control group, ^#^ *p*<0.05 versus with *S-*(-)equol treated group.

To investigate the upstream signaling pathway involved in *S-*(-)equol-mediated upregulation of Nrf2, we tested specific inhibitors of the PI3K/Akt (LY294002) and ER (ICI182, 780) pathways on Nrf2 activation. The dual luciferase reporter assay demonstrated that LY294002 or ICI182,780 significantly abrogated the induction of the NQO1-ARE-dependent firefly luciferase reporter gene activity in response to *S-*(-)equol (Figure 3A). Nuclear Nrf2 has been reported to correlate with total Nrf2. As shown in Figure 3B, in EA.hy926 cells and HUVECs, PI3K/Akt and ER inhibitors impair accumulation of Nrf2, HO-1, or NQO1 in response to *S-*(-)equol or daidzein exposure. In these experiments, NQO1 was the most significantly affected.

To address the role of the Akt and ER pathways in Nrf2 nuclear translocation induced by *S-*(-)equol exposure, we investigated whether inhibitors LY294002 and ICI182,780 affected nuclear translocation of ectopically expressed HA-Nrf2 after *S-*(-)equol treatment. As shown in Figure 3C, in endothelial cells, HA-Nrf2 is predominantly cytoplasmic under steady-state conditions. After *S-*(-)equol treatment, HA translocated to the nucleus, and PI3K/Akt and ER inhibitors blocked *S-*(-)equol-induced HA nuclear accumulation.

Endogenous Nrf2 was measured using immunofluorescence imaging. As shown in Figure 3D, under normal conditions, weak Nrf2 staining was observed in both the cytoplasm and the nucleus, and the nuclear membrane of most cells was poorly defined. In response to *S-*(-)equol and daidzein treatment, Nrf2 obviously accumulated in the nucleus and enhanced signal strength; the nuclear membrane in most cells was readily distinguishable. Inhibitors of PI3K/Akt and ER caused a marked impairment in *S-*(-)equol-induced Nrf2 nuclear translocation. We also studied whether the PI3K/Akt and ER pathways are involved in Nrf2 signaling in response to daidzein treatment. As shown in Figure 3D, treatment with PI3K/Akt inhibitors in combination with daidzein yielded results similar to those observed with *S-*(-)equol. Collectively, these results suggest that *S-*(-)equol-induced Nrf2 nuclear translocation in endothelial cells occurs via the PI3K/Akt and ER pathways.

### ERβ Plays a Role in Activation of Nrf2 by *S*-(-)Equol

**Figure 4:**
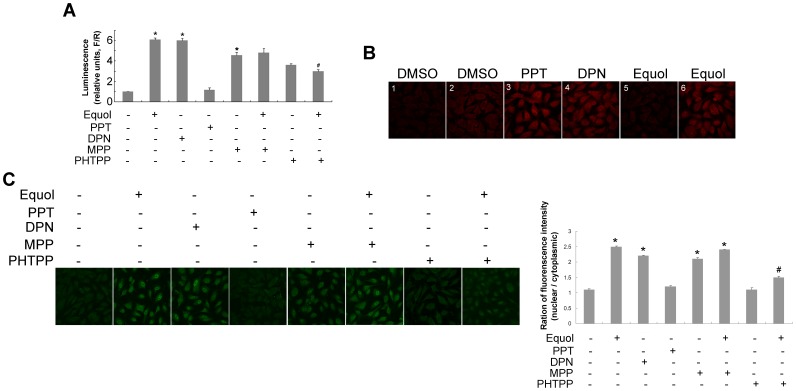
ERβ plays a role in activation of Nrf2 by *S-*(-)equol in endothelial cells. (A) EA.hy926 cells were transfected with ARE-dependent firefly luciferase reporter gene (*F*) and the renilla firefly luciferase gene (*R*), and treated with 100 nM *S-*(-)equol in the presence or absence of 10 nM DPN, 10 nM PPT, 100 nM MPP or 100 nM PHTPP for 2 h following further incubation with or without 100 nM *S-*(-)equol. The potency of induction is expressed as the relative luminescence (R/F) measured using the dual luciferase reporter assay system (mean ± SD, n = 6). *p<0.05 versus with control group, ^#^ *p*<0.05 versus with *S-*(-)equol treated group. (B) EA.hy926 cells were incubated with 0.1% DMSO (1), 10 nM PPT (3), or 100 nM *S-*(-)equol (5) for 16 h and then immunostained with an antibody against ERα; and cells also were incubated with 0.1% DMSO (2), 10 nM PPT (4), or 100 nM *S-*(-)equol (6) for 16 h and then immunostained with an antibody against ERβ, then analyzed by confocal microscopy. (C) Cells were treated with 100 nM *S-*(-)equol in the presence or absence of 10 nM DPN, 10 nM PPT, 100 nM MPP or 100 nM PHTPP for 2 h following further incubation with or without 100 nM *S-*(-)equol, and Nrf2 localization was analyzed by confocal microscopy. Values (intensity of nuclear versus cytoplasmic) are means of counting 100 cells with standard deviations represented by vertical bars. *p<0.05 versus with control group, ^#^ *p*<0.05 versus with *S-*(-)equol treated group.

Two classic ERs exist, ERα and ERβ, and these have somewhat different signaling profiles. Thus, specific activators and inhibitors of both ER subtypes were used to confirm which ER isoform mediated the activation of Nrf2 by *S-*(-)equol. As shown in Figure 4A, similar to *S-*(-)equol, DPN (10 nM) treatment, an ERβ agonist, significantly activated the antioxidant response element in HUVECs transiently transfected with the NQO1-ARE-dependent firefly luciferase plasmid compared with control. However, PPT (10 nM) treatment, to stimulate ERα, did not have this effect. Treatment with PHTPP (100 nM), to antagonize ERβ, significantly decreased luciferase activity induced by *S-*(-)equol compared with the *S-*(-)equol treatment alone group, whereas MPP (100 nM) treatment, to antagonize ERα, had little effect on luciferase expression. Interestingly, MPP treatment alone remarkably increased luciferase activity compared with control.

As shown in Figure 4B1 and 2, both ERα and ERβ were expressed in HUVECs, with weaker expression of ERα (Figure 4B1) than ERβ (Figure 4B2). *S-*(-)equol treatment remarkably upregulated ERβ (Figure 4B6) but not ERα (Figure 4B5) expression compared with positive control which were treated with the specific ERβ activator (Figure 4B4), DPN or the specific ERα activator, PPT (Figure 4B3).

Immunofluorescence revealed the same trend in expression with regard to Nrf2 activation (Figure 4C). DPN (10 nM) treatment notably induced Nrf2 nuclear translocation, whereas PPT (10 nM) failed to do so. PHTPP treatment significantly decreased the fluorescence intensity and nuclear translocation induced by *S-*(-)equol compared with the *S-*(-)equol treatment alone group, however MPP had no effect on inhibition of Nrf2 nuclear accumulation. Similarly, MPP treatment alone enhanced Nrf2 nuclear translocation compared with control, however PHTPP treatment alone had little effect on Nrf2 activation. Data show that *S-*(-)equol activates the Nrf2 transcriptional factor at least in part through ERβ.

### Efficacy of *S*-(-)Equol in Protecting Cells Against Oxidative Stress-induced Damage

**Figure 5:**
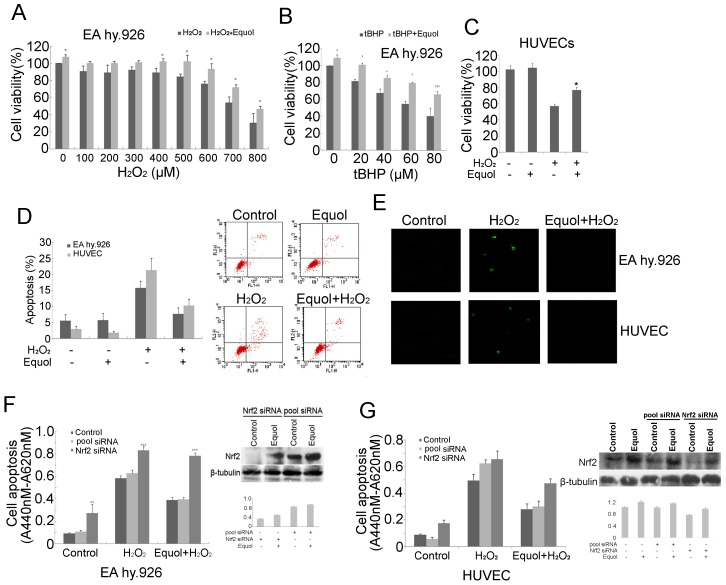
Efficacy of *S-*(-)equol in protecting against oxidative stress-induced toxicity through Nrf2. EA.hy926 cells that were pretreated with 100*S-*(-)equol or sham treated for 24 h were exposed to H_2_O_2_ (100–800 µM) (A) or tBHP (20–80 µM) (B) in the presence or absence of 100 nM *S-*(-)equol for 24 h. HUVECs were pretreated with 100 nM *S-*(-)equol or sham treated for 24 h were exposed to H_2_O_2_ (100 µM) (C). Cell survival after oxidative stress was measured using a CCK-8 assay. The data are reported as the mean ± SD, n = 6. (D) Cells untreated or pretreated with 250 nM *S-*(-)equol for 24 h and then treated with H_2_O_2_ for another 24 h. Apoptotic cell death was detected using Annexin V-FITC and flow cytometry. The mean ± SD was calculated from three independent experiments. (E) Apoptotic cells were visualized by TUNEL. Immunoblot analysis showing Nrf2 protein in EA.hy926 cells (F, right) or HUVECs (G, right) transfected with a control or Nrf2-siRNA. Nrf2 protein was measured with immunoblot analysis using an anti-Nrf2 antibody to confirm knockdown of Nrf2 expression. (F and G) Untransfected cells and cells transfected with indicated siRNAs were incubated with 100 nM *S-*(-)equol for 24 h in the presence of 650 µM H_2_O_2_ or with H_2_O_2_ alone. The apoptotic index for each sample was determined using a cell death ELISA kit. The data are reported as the mean ± SD, n = 3 (*p<0.05, **p<0.01, ***p<0.001 compared with control group, ^#^ *p*<0.05 compared with *S-*(-)equol treated group).

We assessed the effectiveness of *S-*(-)equol in protecting cells from acute cell death in response to oxidative stress induced by H_2_O_2_ or tBHP. Cell death was measured with a CCK-8 assay, which revealed that pre-incubation followed by co-treatment with *S-*(-)equol significantly improved cell survival in response to H_2_O_2_ (Figure 5A and C) and tBHP (Figure 5B) exposure.

Cellular apoptosis was quantified by Annexin V-FITC/flow cytometry. Treatment with 650 µM H_2_O_2_ in EA.hy926 cells or 100 µM H_2_O_2_ in HUVECs for 24 h increased the percentage of apoptotic cells whereas pre-incubation with 250 nM *S-*(-)equol followed by co-treatment with H_2_O_2_ reduced cell death compared to untreated cells (Figure 5D). A TUNEL assay used to detect DNA damage in cells that had undergone apoptosis revealed that positively staining cells increased in response to H_2_O_2_ treatment, and pretreatment with 250 nM *S-*(-)equol reduced positively stained cells in a manner comparable to un-pretreated cells (Figure 5E). The involvement of Nrf2 in *S-*(-)equol-mediated protection against oxidative stress-induced cell death was confirmed by siRNA-induced knockdown of Nrf2. Immunoblot analysis revealed that the typical increase in Nrf2 that occurs in response to *S-*(-)equol did not occur in cells transfected with Nrf2-siRNA in both EA.hy926 cells and HUVECs (Figure 5F right and G right). Nrf2-siRNA transfection significantly increased cell apoptosis of control or H_2_O_2_-treated cells, indicating that Nrf2 has a pronounced effect on cell protection. Moreover, protection against H_2_O_2_-induced apoptosis that is normally conferred by *S-*(-)equol was attenuated in EA.hy926 cell and HUVECs transfected with Nrf2-siRNA (Figure 5F and G), suggesting that Nrf2 activation is a fundamental component of the *S-*(-)equol-induced antioxidant response in endothelial cells.

## Discussion

Here we report that *S-*(-)equol, a metabolite of soy isoflavones, can activate the Nrf2/ARE signaling pathway, a major component of the cellular antioxidant response. This response is mediated by PI3K/Akt and ER pathways, which suggests a specific mechanism by which soy isoflavones protect vascular endothelial cells against oxidative stress.

Recent studies suggest that the Nrf2/ARE signaling pathway may be a promising target for the development of chemopreventive agents to treat atherosclerosis, diabetes, hypertension, and stroke. Several studies suggest that equol is a vasodilator capable of inducing NO-dependent relaxation of endothelial cells [10], [17], [18], [19], [20], [21], [22]. Recent clinical trials also indicate that consumption of soy isoflavone-containing foods significantly improves vasodilatation, decreases arterial stiffness, and lowers diastolic blood pressure [23], [24]. Low doses of *S-*(-)equol can stimulate the Nrf2/ARE pathway, which may suggest a reasonable explanation for the extraordinary antioxidant effect of equol on the cardiovascular system. However, because preclinical data on *S-*(-)equol are scarce, additional mechanistic studies are needed. Such studies, facilitated by a readily available commercial supply of *S-*(-)equol, will enable future clinical investigations of this compound as a pharmaceutical and/or nutraceutical agent.

In this study, we used a well-established luciferase reporter construct, which has proved effective for screening small molecules, including cinnamic aldehyde [15] and oridonin [25]. EA.hy926 cells and HUVECs transiently transfected with the luciferase reporter construct had both a dose- and time-dependent increase in reporter activity in response to *S-*(-)equol. We used low concentrations of *S-*(-)equol (1–250 nM) in our studies. Previous work suggested that 100–500 nM of genistein confers maximal protection against oxidative stress-induced cell death in EA.hy926 cells [26], and evidence suggests that equol is a more active antioxidant and anti-cancer agent than daidzein and genistein. Humans who produce equol (“equol producers”) have plasma equol concentrations as high as 130 nM depending upon their diet [27]. Researchers have also shown that “equol producers” have serum equol and daidzein concentrations in the range of 10.3–139 nM and 16–1,401 nM [28], respectively. Plasma concentrations of genistein range from 40–4,000 nM in these individuals depending on diet [29], [30], [31]. Therefore, understanding the effect of nutritionally relevant plasma equol concentrations (and other soy isoflavones) on cardiovascular cells is essential for understanding the mechanism by which isoflavones function as potential antioxidants.

Many naturally occurring compounds, such as curcumin, sulforaphane, epigallocatechin-3-gallate, caffeic acid phenethyl ester, quercetin, resveratrol and puerarin, have been shown to increase Nrf2 protein [32]. However, limited studies have been published on the relationship between soy isoflavones and Nrf2. *In vivo* studies suggest that diets fortified with genistein (2 g/kg) significantly increased hepatic NQO1 mRNA and activity [33]. In oxidatively stressed endothelial cells, genistein (50 µM) was reported to induce Nrf1-mediated glutathione peroxidase activation without affecting Nrf2 levels [34]. Little direct evidence exists for the activation of Nrf2 by equol. Physiologic concentrations (i.e., 1 and 5 µM) of daidzein and equol, but not genistein, have been shown to increase quinine reductase activity in heap-1c1c7 cells [7]. We observed that treatment with *S-*(-)equol (10–250 nM) causes a dose-dependent increase in Nrf2. These equol doses are much lower than those used in previous experiments, which is consistent with the observations of others that 1–100 nM equol is sufficient to induce rapid vascular relaxation in human endothelial cells [21]. Similar to results obtained after tBHQ treatment, the increase in Nrf2 in response to *S-*(-)equol is due primarily to the stabilization of Nrf2 as opposed to an increase in Nrf2 mRNA. However, we previously found that pretreatment of endothelial cells with 500 nM genistein for 10 h [26] results in a 10-fold increase in Nrf2 mRNA. These results suggest that the activation of Nrf2/ARE-regulated antioxidant genes may occur by increasing Nrf2 protein alone or by increasing Nrf2 protein and mRNA together.

Previous studies show that HUVECs express both ER46 and ER66 [35], whereas EA.hy926 cells exclusively express ER46 [35]. Localization of ERα and ERβ immunoreactivity in HUVECs was first discovered by Joy and co-workers [10]. However, in our study, EA.hy926 cells had weak immunolabelling of both ERα and ERβ under normal conditions, and the intensity of ERβ expression was stronger than that for ERα. Previous work suggests that HUVECs have strong ERβ and weak ERα immunolabelling, and the response of HUVECs to ER modulators was reported to be regulated via ERβ [36]. Additionally, daidzein (100 nM) increased ERβ expression in HUVECs [37] and equol is a known ERβ selectively activator. These results were consistent with our observation that *S-*(-)equol upregulated ERβ but not ERα in EA.hy926 cells.

Equol (as well as daidzein and genistein) itself may positively affect endogenous hormones regulation by binding or dissociating from ERs [38]. Most endothelial effects are ERα mediated and as the effect of equol is really fast, it seems that the membrane bound ER is involved rather than ERβ. But the rapid (several minutes) activation of endothelial NOS and antioxidant defense enzymes by equol involves neither ERα nor ERβ [10]. Nevertheless, ERβ can recruit to the electrophile response element (EpRE) upregulating antioxidative stress enzymes in breast epithelial cell lines [39]. And to our knowledge, only one study suggests that daidzein and equol affect both ERβ and the binding of Nrf2 to the quinine reductase ARE [7], but the relationship between ERβ and Nrf2 is unclear. We found that abrogation of ERβ attenuated the induction of Nrf2 activation by *S-*(-)equol, but that modulation of ERα had no effects on Nrf2. The results to some extent support the previous observations that the regulation of ERβ and antioxidant genes by equol.

Of interesting, in the study, simple repression of ERα significantly increased Nrf2 expression. Most studies with respect to ER and Nrf2 are focused on cancer and one report suggested that inhibition of ERα expression by the antiestrogen shikonin reverses the inhibitory effect of estrogen on NQO1 expression [40]. In addition, the orphan nuclear receptor, estrogen-related receptor beta (ERRβ), which shares a high degree of amino acid identity with ERα, acts as a potent inhibitor of Nrf2 transcriptional activity [41]. Thus, taken together, *S-*(-)equol activates Nrf2 through ER, especially by regulation of ERβ. Of note, suppression of ERα caused the same upregulation of Nrf2, phenomenon which lead us to believe that the selective effect of *S-*(-)equol on cells with different ER isoforms in regulating Nrf2 may be a promising therapeutic target under different pathological conditions.

Activation of the PI3K/Akt pathway plays a role in diverse biological process, including cell proliferation, apoptosis, and survival. PI3K/Akt mediated Nrf2 activation is a well-documented pathway involved in protection against cytotoxicity, including endothelial cell oxidative stress [42], [43]. High concentrations of genistein have a pro-apoptotic effect in cancer cells via stimulation of the PI3K/Akt pathway during tumorigenesis [44], and this work confirmed that the PI3K/Akt/Nrf2 signaling pathway plays a role in *S-*(-)equol mediated cardiovascular cytoprotective effects. Both Nrf2 and eNOS are important for maintaining vascular redox homeostasis. In HUVECs, active Nrf2 was found to help maintain eNOS in naïve and stressed cells [45]. Current studies consistently show that equol rapidly acts upon eNOS synthesis so possible mechanism for equol activity could be activation of membrane bound ER (GPR30) [46], which in turn, activates Akt kinases that phosphorylate eNOS and activate Nrf2 [47].

Equol has a large number of π-electrons, which confers hydrogen donating and free radical scavenging properties. Thus, we tested the antioxidant effects of *S-*(-)equol in response to oxidative stress and found that equol treatment markedly decreased cell apoptosis and increases cell survival. *In vitro* antioxidant properties of equol preparations that were predominantly racemic are well documented [2], [48], [49], [50], [51]. (±)Equol is a more robust antioxidant than any other identified isoflavones. However, as a possible pharmaceutical or nutraceutical agent for a number of hormone-dependent disorders [52], [53], [54], *S-*(-)equol has attracted more interest than *R-*(+)equol, because *S-*(-)equol is made endogenously in the human. Since Nrf2 is important in the attenuation of apoptosis in endothelial cells in response to *S-*(-)equol, whether pure *S-*(-)equol diastereomers such as SE5-OH (natural *S-*equol produced by soy germ fermentation in Lactococcus 20–92) has activity *in vivo* in an atherogenic animal model (ApoE−/− mice) is worthy of study. Finally, to what extent Nrf2 plays a role in the effects of *S-*(-)equol warrants investigation.

## Methods

### Reagents

*S-*(-)equol was obtained from Cayman Chemical Company (Ann Arbor, MI). Daidzein, *tert*-butylhydroquinone (*t*-BHQ), LY294002, ICI182780, DPN, PPT, MPP, PHTPP, hydrogen peroxide (H_2_O_2_) and *tert*-butyl hydroperoxide (*t*-BHP) were purchased from Sigma-Aldrich (St. Louis, MO). The Nrf2 (sc-722) primary antibody used for Western blot analysis, Nrf2 siRNA (sc-37030), control siRNA (sc-37007) and siRNA transfection reagents (sc-29528) were obtained from Santa Cruz Biotechnology (Santa Cruz, CA). The Nrf2 primary antibody used for the immunocytochemistry analyses was purchased from Abcam (Cambridge, UK). HA antibody was obtained from Thermo Scientific (Thermo, CA). NQO1 antibody (A180) was purchased from NOVUS Biologicals (Littleton, CO). HO-1 (SPA-895) antibody was obtained from Stressgen (Farmingdale, NY). ERα (BS1535) and ERβ (BS2429) antibody and β-tubulin (BS1482) antibody were purchased from Bioworld Technology (Louis Park, MN). Goat anti-rabbit and goat anti-rabbit IgG peroxidase conjugates were obtained from Pierce Biotechnology (Rockford, IL). The Alex Fluor 594-labled Goat Anti-Rabbit IgG (H+L) was from the Beyotime Institute of Biotechnology (Beyotime, Jiangsu, China). The NQO1-ARE-luciferase reporter gene and HA-expression plasmids were kindly provided by Dr. Donna D. Zhang (University of Arizona, Tucson).

### Cell Culture

HUVEC investigations conformed with the principles outlined in the Declaration of Helsinki, and all protocols were approved by the Third Military Medical University ethics review board. Primary HUVECs were isolated by infusion with PBS buffered salt solution containing 0.25% (w/v) pancreatic enzyme and incubated for 5 min at 37°C, and this procedure was repeated once. The secreted solution containing pancreatic enzyme and cells was centrifuged (1,500 rpm) for 10 min and the cell pellet was then resuspended and cultured in Medium 199 supplemented with 10% heat-inactivated fetal bovine serum, 2 mM L-glutamine, 30 µg/ml ECGS, 20 µg/ml heparin, phenol red, 100 U/ml penicillin, and 100 µg/ml streptomycin. Cells were cultured in a 5% CO_2_ incubator and used at passage 2 to 4. Cells were seeded into cell culture plates and cultured overnight to allow cells to attach before further treatment. The HUVEC line EA.hy926 was obtained from American Type Culture Collection (ATCC). Cells were maintained at 37°C in an incubator with a humidified atmosphere of 5% CO_2_ and were cultured in Dulbecco’s Modified Eagle’s Medium (DMEM)/High Glucose supplemented with 10% heat-inactivated fetal bovine serum, 4,500 mg/L glucose, 4 mM L-glutamine, phenol red, 100 U/ml penicillin, and 100 µg/ml streptomycin. Cells were grown to 90–95% confluence and then diluted into fresh medium.

### Luciferase Reporter Gene Assay

The cells were plated in 24-well plates and co-transfected with an ARE-luciferase plasmid and a renilla luciferase control plasmid and pGL 4.74 [hRluc/TK] (Promega, Madison, WI). The transfections were performed using Lipofectamine™ 2000 (Invitrogen, Carlsbad, CA). Transfected cells were treated with equol for 10 h, and firefly and renilla luciferase activity was measured using the dual luciferase reporter assay system from Promega.

### Western Blot Analysis

The treated cells were lysed in lysis buffer (50 mM Tris pH 8.0, 150 mM NaCl, 0.1% SDS, 1% Triton X-100, 0.5% deoxycholate and protease inhibitors), and protein concentrations of the lysates were measured using the Bradford assay. The lysates (40 µg/well) were resolved on 10–12% SD*S-*polyacrylamide gels and transferred to a PVDF membrane. Membranes were incubated with primary antibody overnight at 4°C, washed with PBS, and then incubated with the appropriate horseradish peroxidase (HRP)-conjugated secondary antibody (1∶5,000 dilution). Immune complexes were visualized by enhanced chemiluminescence followed by exposure to X-ray film.

### Real-time PCR

Total RNA was extracted using Trizol reagent (Bioer, Hangzhou, China), and the RNA preparations were cleared of contaminating genomic DNA by DNase treatment. Reverse transcription was performed using M-MLV reverse transcriptase (Bioer, China). Gene expression was measured by quantitative real-time PCR using a SYBR Green PCR kit (Bioer, Hangzhou, China). The primers used for expression analysis were as follows: Nrf2, 5′-ATTGCCTGTAAGTCCTGGTCA-3′ (forward) and 5′-ACTGCTCTTTGGACATCATTTCG-3′ (reverse); HO-1, 5′-CGATGGGTCCTTACACTC-3′ (forward) and 5′-GGCTCCTTCCTCCTTT-3′ (reverse); NQO1, 5′-AAACCTCCTTTACCAG-3′ (forward) and 5′-GAAGATGAAGGCAACA-3′ (reverse); GAPDH, 5′-TGCACCACCAACTGCTTAG-3′ (forward) and 5′-GATGCAGGGATGATGTTC-3′ (reverse); 18S rRNA, 5′-GTAACCCGTTGAACCCCATT-3′ (forward) and 5′-CCAT CCAATCGGTAGTAGCG-3′ (reverse); and β-actin, 5′-ACCAACTGGGACGATATGGAGAAGA-3′ (forward) and 5′-ACGACCAGAGGCATACAGGGACAA-3′ (reverse). Real-time PCR conditions were as follows: 94°C for 2 min followed by 45 cycles of 94°C for 10 s and 72°C for 45 s. Data are presented as the fold-change in gene expression relative to the control group.

### Immunofluorescence

Cells were seeded on sterile glass coverslips and left to attach overnight. After treatment, the cells were fixed with 4% paraformaldehyde and washed 3 times with PBS. The cells were subsequently permeabilized with 1% Triton X-100 for 5 min, and then washed and blocked with BSA (1%). After incubation with the primary antibody, the coverslips were washed and incubated with the appropriate FITC-conjugated goat anti-rabbit secondary antibody (1∶100 dilution, Zhongshan, China) for 2 h at 37°C. After 3 more washes with PBS, cells were counterstained with 1 µg/ml of DAPI for 5 min. Finally, cells were mounted on slides with mounting medium (Dako, Hamburg, Germany) and analyzed by confocal laser scanning microscopy.

### Measurement of Cell Viability

Cell viability was assessed using a CCK-8 assay (Cell Counting Kit-8, Dojindo Laboratories, Japan). WST-8 [2-(2-methoxy-4-nitrophenyl)-3-(4-nitrophenyl) -5-(2,4-disulphophenyl)-2H-tetrazolium, monosodium salt] is reduced by cellular dehydrogenases to yield a water-soluble, orange dye. The relative amount of dye formed is directly proportional to the number of living cells. For this assay, the cells were seeded at a density of 5,000 cells/well in 96-well plates, with six replicate wells for each condition on the same plate. The CCK-8 reagent was diluted ten-fold with DMEM before being added (100 µl) to each well. Two-and-a-half hours later, sample ODs were read at 450 nm using a multimode microplate reader (Infinite M200, Tecan, Switzerland). The OD_450_ is proportional to the degree of cell viability. Data shown represent the mean of at least three independent experiments.

### Flow Cytometry Assay

Cells grown in 6-well plates were harvested, washed, double-stained with Annexin V-FITC and propidium iodide (Annexin V-FITC apoptosis kit, Bestbio, Hangzhou, China), incubated for 15 min at room temperature in the dark, and analysed by flow cytometry.

### TUNEL Assay

Apoptosis was detected via terminal deoxynucleotidyl transferase-mediated dUTP-biotin nick-end labelling (TUNEL) analysis using the *in situ* cell death detection kit (Roche, Germany) according to the manufacturer’s instructions.

### siRNA Transfection

Nrf2 siRNA transfections were performed according to the manufacturer’s instructions. Cells were seeded in a 6-well tissue culture plate (2×10^5^ cells per well) in 2 ml antibiotic-free normal growth medium supplemented with FBS and incubated at 37°C in a CO_2_ incubator overnight. Mixtures containing 6 µl of the Nrf2 siRNA and 6 µl of siRNA transfection reagent were incubated for 45 min at room temperature and then added to the cells along with antibiotic- and serum-free medium. The final Nrf2 siRNA concentration was 60 nM. Cells transfected with the control siRNA were treated in parallel.

### Cell Apoptosis Assay

The extent of DNA fragmentation within apoptotic cells was determined using the Cell Death Detection ELISA^plus^ kit (Roche, Germany), which measures cytoplasmic histone-associated DNA fragments using antibodies against biotinylated histones and DNA-POD.

### Statistical Analysis

Data are expressed as means and standard deviations. Statistical significance was analyzed via ANOVA, and differences among groups were assessed via Tukey’s test using SPSS version 13.0 software (SPSS, Inc.). The Student’s *t* test was also used when comparing the means of two groups. Differences were considered statistically significant at *p*<0.05.
